# Structure and Function of a Novel Cellulase 5 from Sugarcane Soil Metagenome

**DOI:** 10.1371/journal.pone.0083635

**Published:** 2013-12-17

**Authors:** Thabata M. Alvarez, Joice H. Paiva, Diego M. Ruiz, João Paulo L. F. Cairo, Isabela O. Pereira, Douglas A. A. Paixão, Rodrigo F. de Almeida, Celisa C. C. Tonoli, Roberto Ruller, Camila R. Santos, Fabio M. Squina, Mario T. Murakami

**Affiliations:** 1 Laboratório Nacional de Ciência e Tecnologia do Bioetanol (CTBE), Centro Nacional de Pesquisa em Energia e Materiais, Campinas, São Paulo, Brazil; 2 Laboratório Nacional de Biociências (LNBio), Centro Nacional de Pesquisa em Energia e Materiais, Campinas, São Paulo, Brazil; University of Waikato, New Zealand

## Abstract

Cellulases play a key role in enzymatic routes for degradation of plant cell-wall polysaccharides into simple and economically-relevant sugars. However, their low performance on complex substrates and reduced stability under industrial conditions remain the main obstacle for the large-scale production of cellulose-derived products and biofuels. Thus, in this study a novel cellulase with unusual catalytic properties from sugarcane soil metagenome (CelE1) was isolated and characterized. The polypeptide deduced from the *celE1* gene encodes a unique glycoside hydrolase domain belonging to GH5 family. The recombinant enzyme was active on both carboxymethyl cellulose and β-glucan with an endo-acting mode according to capillary electrophoretic analysis of cleavage products. CelE1 showed optimum hydrolytic activity at pH 7.0 and 50 °C with remarkable activity at alkaline conditions that is attractive for industrial applications in which conventional acidic cellulases are not suitable. Moreover, its three-dimensional structure was determined at 1.8 Å resolution that allowed the identification of an insertion of eight residues in the β8-α8 loop of the catalytic domain of CelE1, which is not conserved in its psychrophilic orthologs. This 8-residue-long segment is a prominent and distinguishing feature of thermotolerant cellulases 5 suggesting that it might be involved with thermal stability. Based on its unconventional characteristics, CelE1 could be potentially employed in biotechnological processes that require thermotolerant and alkaline cellulases.

## Introduction

In the face of growing energy costs, dwindling fossil resources, environmental pollution and a globalized economy, the large-scale use of biotechnology instead of, or to complement, traditional industrial production processes, particularly in the chemical sector, is viewed as both an opportunity and a necessity to a more social and ecological sustainable energetic matrix [1]. In this context, metagenomics has received much attention owing to its great potential to provide new biocatalysts with diverse functions and applications. To illustrate that, a soil sample might contain in the order of 10^4^ different bacterial species and more than one million novel open reading frames, many of which encode putative enzymes [1]. This approach has been broadly employed to find new enzymes to assist ethanol production through lignocellulosic biomass degradation thereby enabling a new generation of biofuels production [2]. 

The current scenario for the reduction of biomass into fermentable sugars via enzymatic routes is characterized by very scanty exemplars of glycoside hydrolases suitable for this process in an industrial scale and rate. Thus, the discovery of new enzymes with higher catalytic efficiency and stability under industrial conditions, and even specialized for the different biomass sources, such as sugar-cane bagasse, may have a revolutionary role in making biofuel production from plant biomass economically viable [3,4]. It is more pronounced when focused on cellulose degradation since cellulases are considered the main bottleneck in biomass breakdown, principally due to very low catalytic efficiency. Moreover, cellulose is the major polysaccharide found in vegetal biomass and plants produce about 180 billion tons of cellulose per year globally, making this carbohydrate the largest organic carbon reservoir on earth [5]. Consequently, efficient breakdown of cellulose is an essential pre-requisite for the production of biofuels and cellulases are key enzymes in this process [6]. The catalytic domains of cellulases are found in 14 families of glycoside hydrolases that have been classified according to their sequence [7]. Cellulases or endoglucanases (EC 3.2.1.4) catalyze the cleavage of random internal β-1,4-glycosidic bonds in cellulosic chains and can be found coupled to the cellulosome or generally secreted as independent enzymes. Very often, the catalytic domain of cellulases is associated to one or more carbohydrate-binding modules, which binds to the substrate and increase the catalytic efficiency of some enzymes.

Here, we report a novel cellulase belonging to GH5 family, named as CelE1, retrieved from a sugarcane soil metagenomic library, which is a promising biocatalyst in biofuels production. The thorough biochemical and structural characterization, providing details about three-dimensional structure, catalytic properties and stability of CelE1, might contribute to broaden our understanding of the molecular basis for glycoside hydrolases adaptation to use surgacane biomass as substrate and then serve as an instrumental model for enzyme redesign and optimization aiming at vegetal biomass degradation.

## Materials and Methods

### Identification, cloning and sequencing of the CelE1 gene

To isolate cellulolytic clones, a functional screening of a metagenomic library derived from sugarcane field land soil was performed [8]. Briefly, recombinant *E. coli* cells were spread on plates containing 0.5% (w/v) carboxymethyl cellulose (CMC) as substrate and colonies producing clear hydrolytic halos were selected by staining with Congo red [9]. 

 The plasmid was extracted from the positive clone and sequenced using universal M13 forward and reverse primers on an ABI Prism 377 Genetic Analyzer (Applied Biosystems, USA) at the Brazilian Bioethanol Science and Technology Laboratory. The sequence was analyzed using Geneious Pro 4.8.5 and the identified ORF was compared to public databases available at NCBI by BLASTx tool. The nucleotide sequence of CelE1 gene was deposited in the GenBank database under the accession number KF498957. 

### Protein expression and purification of the catalytic domain

The coding region corresponding to the GH5 catalytic domain of CelE1 was amplified by PCR using primers containing *Nde*I (5’-TATATATCATATGGTCGCACCCATTACTACCAGC-3’) and *Bam*HI (5’-ATAGGATCCTTACGGCCAACCGGAAATAAT-3’) restriction sites (underlined) for cloning into the plasmid expression vector pET-28a(+) (Novagen). 

The plasmid pET28a harboring the encoding sequence of *celE1* was transformed in *E. coli* Rosetta-gami™(DE3)pLys cells and the protein expression performed under standard conditions at 37 °C for 4 hours in selective LB medium (kanamycin) containing 0.5 mM IPTG (isopropyl β-D-thiogalactopyranoside). The cells were harvested, resuspended in lysis buffer (20 mM sodium phosphate pH 7.4, 100 mM NaCl, 5 mM benzamidine, 1 mM PMSF) and then sonicated with 6 pulses of 30 s at 500 W, using a VC750 Ultrasonic Processor (Sonics Vibracell). The lysate was clarified (20,000 xg for 30 min) and the supernatant was loaded onto a nickel-affinity column (GE Healthcare) which was washed and the recombinant protein eluted using a non-linear gradient of imidazole (0 to 500 mM). The fractions were then pooled, concentrated and subsequently applied to a size-exclusion chromatography column (Superdex 75, GE Healthcare), pre-equilibrated with 50 mM sodium phosphate buffer pH 7.4, 150 mM NaCl. The sample purity was confirmed by polyacrylamide gel electrophoresis under denaturing conditions [10]. Protein concentration was estimated by absorbance at 280 nm using molar extinction coefficient for the polypeptide deduced from gene (http://web.expasy.org/protparam/). 

### Enzyme characterization and cellulase activity

The hydrolytic activity was determined by quantifying the amount of reducing sugar released from different polysaccharides using the 3,5–dinitrosalicylic acid method [11]. One unit (U) was defined as the quantity of enzyme required to release 1 μmol of reducing sugar per min. To determine the optimum pH, the enzymatic reactions were carried out in 200 mM phosphate-citric acid-glycine buffer (pH 2 - 12) containing 0.5% (w/v) CMC and, incubated at 37 °C during 20 min. For optimal temperature determination, the reactions were in the same buffer (pH 7.0) but incubations were in the range of 15 - 90 °C. 

Experimental conditions (reaction time and enzymatic units) were adjusted to guarantee the estimation of initial velocities (hydrolysis of no more than 5% initial concentration of substrate to obtain linear response of product formation in respect to reaction time) for the determination of kinetic parameters K_m_, *V*_max_ and *k*_cat_. Assays were conducted in 50 mM phosphate buffer pH 7.0 at 50 °C during 15 min using 0.06-5.4% (w/v) CMC as substrate. Due to the natural heterogeneity of the substrate, the apparent affinity constant K_m_ was expressed as mg.ml^-1^. Mathematical adjustments were made using the software Graph Pad Prism 5.0 (GraphPad Software) to calculate parameters. The assays were performed in quintuplicate. 

The substrate specificity was evaluated against a set of natural polysaccharides at 37 °C using McIlvaine buffer (pH 6) during 30 min [12]. Hydrolytic activity over industrially-relevant insoluble substrates including pretreated sugarcane bagasse (BEX) (a generous gift from Prof. Dr. George Jackson) and Avicel PH-101 (from Sigma USA) was also tested. In these experiments, a reaction containing 1.0% of each substrate in 200 mM sodium phosphate buffer pH 7.5 were incubated with 10 µg of recombinant protein at 50 °C during 200 min and 40 °C during 24 h, under constant agitation (1,000 rpm). The released sugars were separated from residual polysaccharide by centrifugation 10,000 xg for 20 min and quantified onto supernatant. 

### Capillary electrophoresis of oligosaccharides

Cellohexaose (from Megazyme) and oligosaccharides released by enzymatic activity were derivatized with 8-aminopyrene-1,3,6-trisulfonic acid (APTS) by reductive amination. Enzymatic reactions were performed as described previously, using cellohexaose a substrate [13]. Capillary zone electrophoresis of oligosaccharides was performed on a P/ACE MQD instrument (Beckman Coulter) equipped with laser-induced fluorescence detection. A fused-silica capillary (TSP050375, Polymicro Technologies) of internal diameter of 50 μm and total length of 31 cm was used as separation column for oligosaccharides. Electrophoresis conditions were 15 kV/70–100 μA at a controlled temperature of 20 °C. Oligomers labeled with APTS were excited at 488 nm and emission was collected through a 520 nm band pass filter.

### Circular dichroism spectroscopy and thermal unfolding studies

Far-UV circular dichroism (CD) spectra of CelE1 (20 mM phosphate buffer, pH 7.4 at 25 °C) was measured in the range 195-260 nm in a Jasco J-810 spectropolarimeter (Jasco International Co.) coupled to a Peltier temperature controller using a 1 mm quartz cuvette. Protein concentration was set to 10 μM and the results were expressed as mean residue ellipticity (deg.cm^2^.dmol^−1^.residue^−1^). A total of 30 spectra were collected, averaged and corrected by subtraction of the blank. In order to investigate the thermal stability, CD spectra were analyzed at different temperatures ranging from 20 to 100 °C. Thermal unfolding was monitored by CD intensity changes at 222 nm. Data deconvolution for prediction of secondary structure was performed in DichroWeb server [14,15].

### Crystallization

Crystallization experiments were performed by the sitting-drop vapor-diffusion method using a Cartesian HoneyBee 963 system (Genomic Solutions) and a protein concentration of 17.5 mg.ml^-1^. 544 different formulations based on commercial crystallization screens were tested including those from Hampton Research (SaltRX, Crystal Screen and Crystal Screen 2), Emerald BioSystems (Precipitant Synergy and Wizard I and II) and Qiagen/NeXtal (PACT and JCSG+). Sitting drops were prepared by mixing 0.5 μl of the protein solution with an equal volume of mother liquor and equilibrated against 80 μl of the reservoir solution at 18 °C. Small and clustered crystals were obtained from the condition consisting of 35% (v/v) isopropanol, 30% (w/v) PEG3350 and 100 mM Tris-HCl pH 8.5. The condition was refined varying the isopropanol concentration and by adding glycerol to prevent crystal clustering. Best crystals were grown in 7 days from the condition 30% (v/v) isopropanol, 30% (w/v) PEG3350, 100 mM Tris-HCl pH 8.5 and 5% (v/v) glycerol as additive.

### Data collection and processing

X-ray diffraction data were collected on W01B-MX2 beamline at the Brazilian Synchrotron Light Laboratory (Campinas, Brazil). The wavelength was set to 1.459 Å and the intensities were recorded in a Mar Mosaic 225 mm charged-coupled device detector. The complete data set were collected to a maximum resolution of 1.78 Å. Data were indexed, integrated, merged and scaled using the HKL2000 package (Otwinowski and Minor, 1997). The reflections were indexed in the monoclinic crystal system with unit-cell parameters a= 41.88, b=87.51 c=66.63 Å. An examination of the systematic absences indicated that the crystal belonged to space group P2_1_. Calculation of the Matthews coefficient [16] based on the molecular weight of 32,186 Da (monomer) resulted in a V_M_ of 1.88 Å^3^.Da^-1^ and a solvent content of 34.48%, which corresponds to the presence of two molecules per asymmetric unit. The statistics of the data processing are summarized in Table 1.

**Table 1:** Crystal data and refinement statistics for CelE1.

**Parameter**	
PDB code	4M1R
**Data collection**	
Space group	*P*12_1_1
*Cell dimensions*	
*a*, *b*, *c* (Å)	41.88, 87.51, 66.63
α, β, γ (°)	90, 98.55, 90
Resolution (Å)	50.00 - 1.80 (1.86-1.80)
*R*_merge_	9.6 (30.2)
*<I* / σ*I*>	16.9 (3.9)
Completeness (%)	93.5 (80.4)
Multiplicity	6.5 (3.8)
**Refinement**	
*R*_work_ / *R*_free_ (%)	13.33/ 17.34
Protein molecules in ASU	2
Ligand/ion	2
Water molecules	379
Mean *B*-factor (Å^2^)	12.9
*R.m.s. deviations*	
Bond lengths (Å)	0.024
Bond angles (°)	1.676
*Ramachandran Plot*	
Favored (%)	96.3
Allowed (%)	3.7

Values in parentheses are for highest – resolution shell.

### Structure determination and refinement

The structure of CelE1 was solved by molecular replacement using the program MOLREP [17] and a model based on the atomic coordinates of a psychrophilic cellulase from *Pseudoalterromonas halaplanktis* (PDB code 1TVP, [18]), which displays 67% sequence identity with CelE1. Two clear solutions were observed, which confirmed the presence of two molecules in the asymmetric unit and the initial model after rigid-body refinement resulted in R_factor_/R_free_ of 44/48%. Structure refinement was carried out with the program REFMAC5 [19]. After each cycle of refinement, the model was inspected and manually adjusted into the (2F_o_-F_c_) and (F_o_-F_c_) electron density maps using the program COOT [20]. In the later cycles, water molecules and ligands were added manually and refined. The atomic coordinates and structure factors of CelE1 have been deposited in the Protein Data Bank under the accession code 4M1R.

## Results and Discussion

### Isolation and identification of a novel cellulolytic gene from sugarcane soil metagenome

In order to obtain new cellulolytic enzymes, functional screening of a metagenomic library using CMC as substrate was carried out. One clone of 3100 bp displaying cellulase activity was isolated and sequenced which included the complete CelE1-encoding open reading frame. *CelE1* is 1284 bp long and encodes a polypeptide of 428 amino acid residues containing a putative N-terminal signal peptide followed by a glycoside hydrolase domain belonging to family 5 (GH5). 

The polypeptide corresponding to the GH5 domain of the enzyme shares high sequence identity to previously characterized glucanases including the endoglucanase from *Cellvibrio japonicus* Ueda107 (80% identity, GenBank accession number YP_001983438.1), a cellulase isolated from a bacterium enrichment culture (75% identity, GenBank accession number ACR23656.1) and an endoglucanase from an uncultured organism (74% identity, GenBank accession number ACY24859.1). These analyses confirm that CelE1 belongs to the family 5 of glycoside hydrolases.

### CelE1 displays unusual pH dependence for catalysis

**Figure 1:**
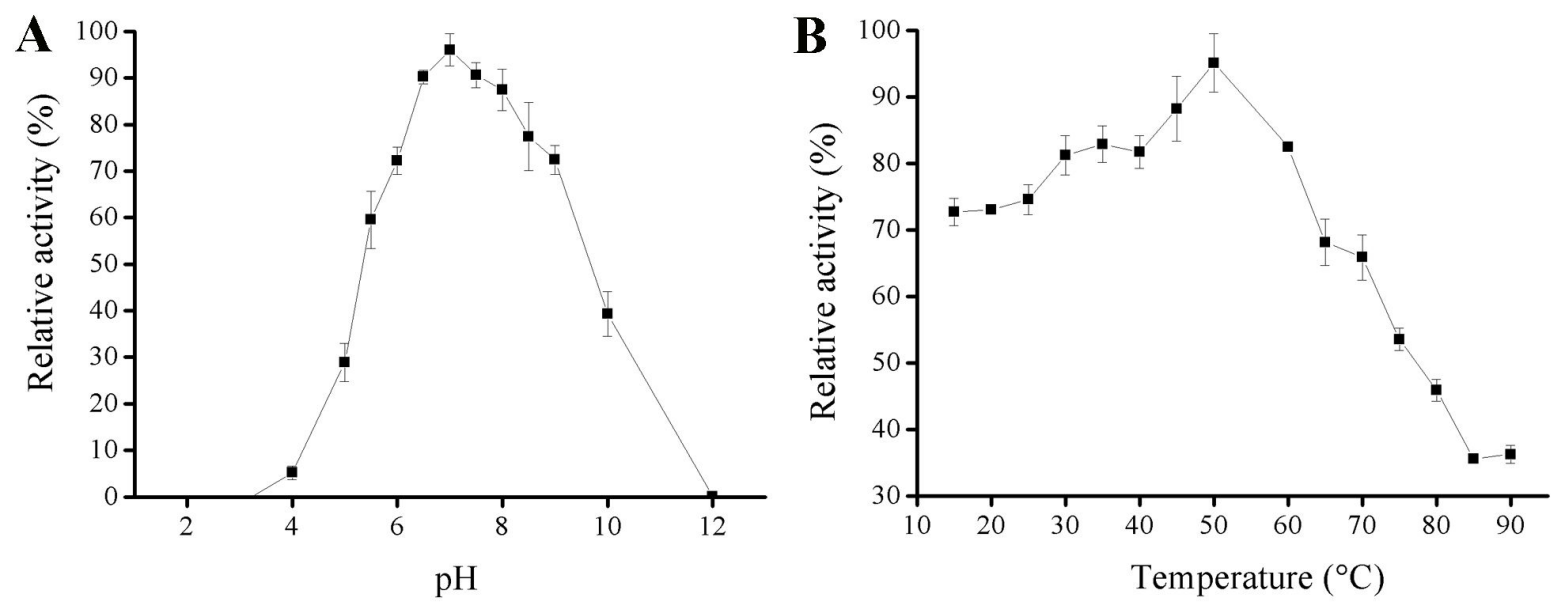
Effect of pH and temperature on the hydrolytic activity of CelE1. Enzyme was incubated in 200 mM phosphate-citric acid-glycine buffer containing 0.5% (w/v) of CMC as substrate for 20 min and the amount of reducing sugars measured by the 3,5–dinitrosalicylic acid method. (A) Measurements were carried out in pH values ranging from 2 to 12 by incubation at 37 °C. (B) Activity assayed under different temperatures at pH 7. Assays were performed on quadruplicate aliquots. Each experiment was repeated three times.

To obtain the catalytic domain and advance with the biochemical and structural characterization, the coding region of CelE1 was sub-cloned into pET28a(+) vector, produced in *E. coli* cells and purified to homogeneity. Recombinant CelE1 was active (relative activity > 30%) over a broad pH range from 5 to 10 with maximum enzyme activity at neutral pH (Figure 1A). In addition, the enzyme retains 70% of the activity in pH values of 8 and 9. Usually cellulases belonging to GH5 family display an optimum for activity at slightly acid pH as observed for the hyperthermophilic CelB from *Caldicellulosiruptor saccharolyticus* (pH 5.5) [21], Cel5G derived from a soil metagenomic library (pH 4.8) [22], BsCel5A from *Bacillus subtilis* (pH 6) [23] and cellulase 5 from the buffalo rumen metagenomic library (pH 5.5) [24]. However, this novel cellulase exhibited a bell-shaped curve for pH dependence, shifted towards basic conditions, which can be attractive for biotechnological applications in which conventional acidic cellulases are not suitable.

Furthermore, the effect of temperature on activity of CelE1 was examined by monitoring the hydrolysis of CMC in the range from 10 to 90 °C at pH 7 (Figure 1B). The enzyme activity increased in a temperature–dependent manner reaching a maximum at 50 °C as described for other characterized cellulases [23-26]. Interestingly, the enzyme showed more than 60% of the relative activity at temperatures around to 70 °C and higher levels even at low temperatures (10 - 50 °C) indicating that CelE1 could be considered as a thermotolerant enzyme with significant catalytic activity ( > 65%) at a broad temperature range from 10 to 70 °C. 

 The apparent kinetic parameters were determined in the presence of different concentrations of CMC resulting in K_m_ and *k*_cat_ values of 6.05 ± 0.37 mg.ml^-1^ and 24.54 s^-1^, respectively. Based on these values the catalytic efficiency was 4.06 mg.ml^−1^s^−1^ (*k*_cat_/K_m_) that is similar to that observed for other glycoside hydrolases such as *Thermoanaerobacter tengcongensis* cellulase [27], cellulase 5A from *Bacillus subtilis* [23] and the cellulase 5A from *Clostridium thermocellum* [28]. Altogether, the catalytic efficiency of CelE1 combined with the ability to be active in alkaline pHs and in a broad range of temperature, suggest a potential application of this enzyme for industrial processes involving cellulose degradation.

### CelE1 is an endo-acting cellulase with high activity on complex and industrially-relevant polysaccharides

**Figure 2:**
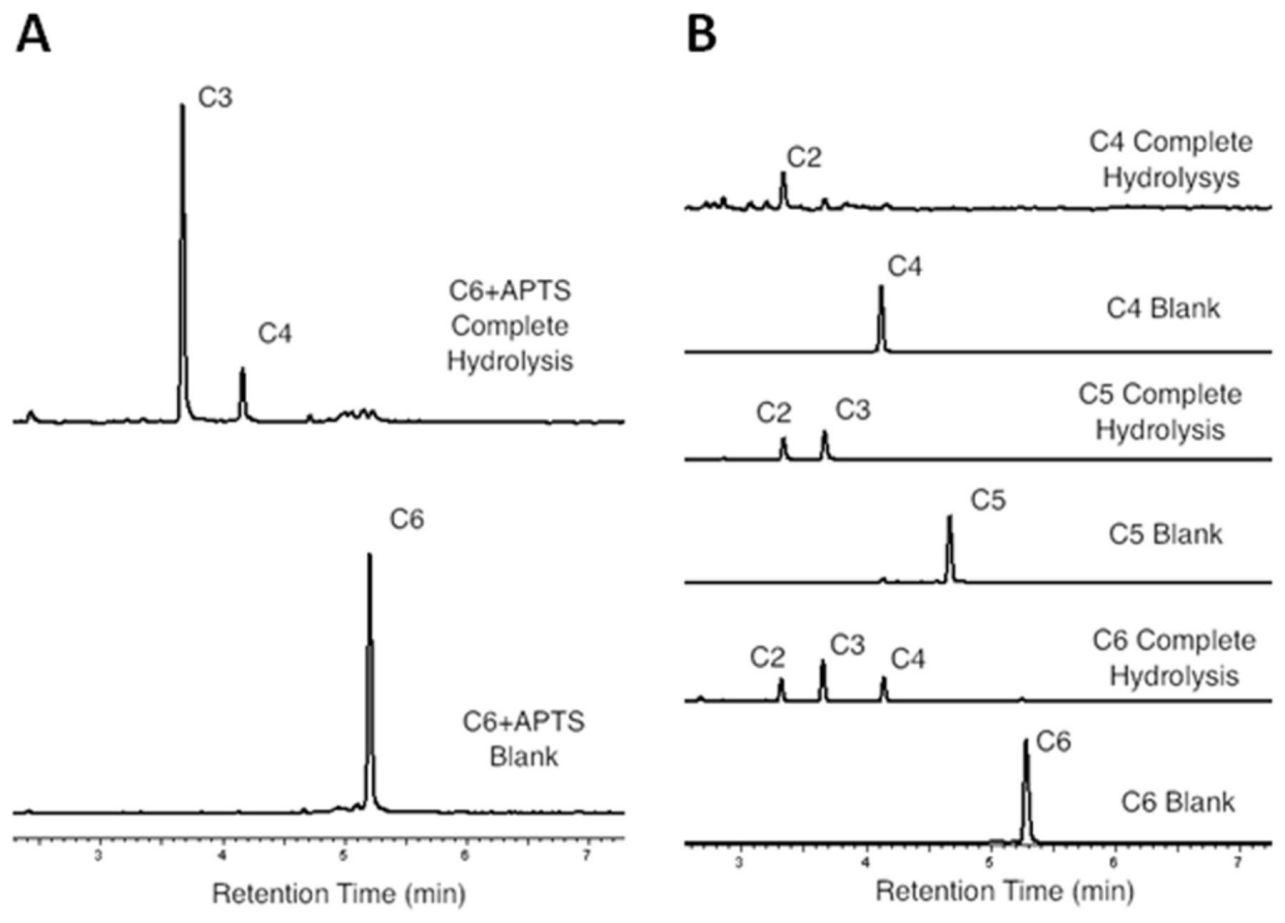
Cleavage pattern of CelE1 on different cellooligosaccharides (cellotetraose (C4), cellopentaose (C5) or cellohexaose (C6)) indicating a classical endo-acting mode. (A) Capillary-zone-electropherogram of the APTS-labeled-cellohexaose hydrolysis (substrate). (B) Analysis of APTS-labeled products of C4, C5 and C6 hydrolysis.

In order to define the action mode for CelE1, capillary zone electrophoresis experiments were carried out with cellohexaose (Figure 2). The cleavage pattern with the predominance of cellobiose, cellotriose and cellotetraose as final products in an oligosaccharide-length manner indicates that the enzyme most likely attacks the internal glycosidic linkages. It confirms that CelE1 is an endoglucanase with action mode similar to the cellulase GH5 from *Erwinia chrysanthemi* [29]. 

Moreover, the substrate specificity of CelE1 was assessed by measuring the hydrolytic activity against eight carbohydrates. Table 2 shows that CelE1 was able to degrade efficiently CMC and β-glucan, the latter being most efficiently hydrolyzed. To further examine the ability of this cellulase to breakdown insoluble complex carbohydrates with industrial interest, hydrolytic activity was investigated using Avicel and BEX as substrates. The rate of sugar released using Avicel was 2- and 8-fold higher than obtained for cellulases Cel5A from *Gloeophyllum trabeum* and endoglucanase CgEG1 from *Coptotermes gestroi*, which were 4.5 x 10^-3^ and 1.1 x 10^-3^ U.mg^-1^, respectively [30,31]. The glycoside hydrolase CelE1 was also active on BEX (Table 2). These findings make CelE1 a promising candidate with potential applicability in industrial processes involving the deconstruction of plant biomass.

**Table 2:** Substrate specificity of CelE1.

**Substrate**	**Specific activity (U/mg)**		
***Simple carbohydrates***			
CMC	13.1	±	1.2
β-glucan	38.8	±	4.4
Galactomannan	0.0	±	0.0
Xyloglucan	0.0	±	0.0
Xylan from Beechwood	0.0	±	0.0
Rye Arabinoxylan	0.0	±	0.0
Sigmacell cellulose (type 20)	2.0	±	0.6
Pectin	0.4	±	0.02
***Complex carbohydrates***			
Avicel	(8.8	±	0.3) x 10^-3^
BEX	(10.2	±	0.1) x 10^-3^

### Structural stability of CelE1

**Figure 3:**
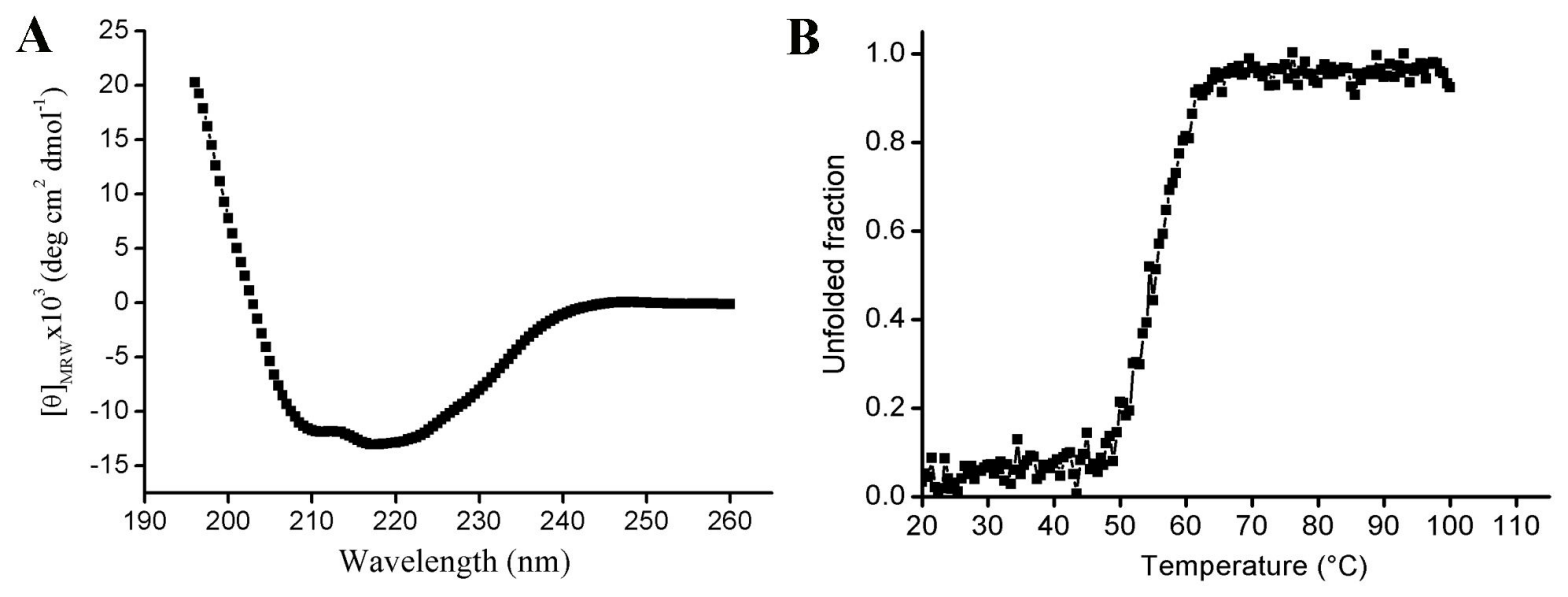
Biophysical characterization of CelE1. (A) Far-UV CD spectrum of CelE1 with typical profile of α/β proteins. (B) Thermal denaturation profile characterized by a single transition and a melting temperature of 55 °C.

 CD analysis of CelE1 resulted in a typical far-UV spectrum of α/β proteins as expected for a GH5 family member indicating a native-like structure of the recombinant enzyme (Figure 3A). In order to investigate its thermal stability, unfolding experiments were performed (Figure 3B). The enzyme presented a melting temperature (T_M_) of 55 °C, which is in agreement with the temperature for maximum catalytic activity (Figure 1B). Interestingly, CelE1 showed significant higher thermal stability than its psychrophilic orthologs such as Cel5G from *Pseudoalteromonas haloplanktis* (T_M_ = 43 °C), despite high sequence identity (67%) [32]. Thus, high-resolution structural data combined with extensive comparative analysis could provide insights into the molecular basis for structural stability differentiation among highly similar cellulases 5.

### Crystallographic depiction of CelE1 at high resolution

The structure of this novel cellulase 5 was determined by X-ray crystallography at 1.8 Å resolution. The crystals belonged to the monoclinic space group P2_1_ with two molecules in the asymmetric unit (Table 1), which could suggest a quaternary arrangement. However, analysis of crystalline contacts using the PDBePISA server [33] did not indicate the presence of any stable protein–protein interface discarding the existence of CelE1 as a dimer in solution. The monomeric form was also confirmed by dynamic light scattering (DLS) measurements (Figure S1 in File S1) and analytical size-exclusion chromatography (Figure S2 in File S1). DLS measurements resulted in a hydrodynamic radius of 2.8 nm (20.6% polydispersity) that corresponds to the monomer of CelE1.

**Figure 4:**
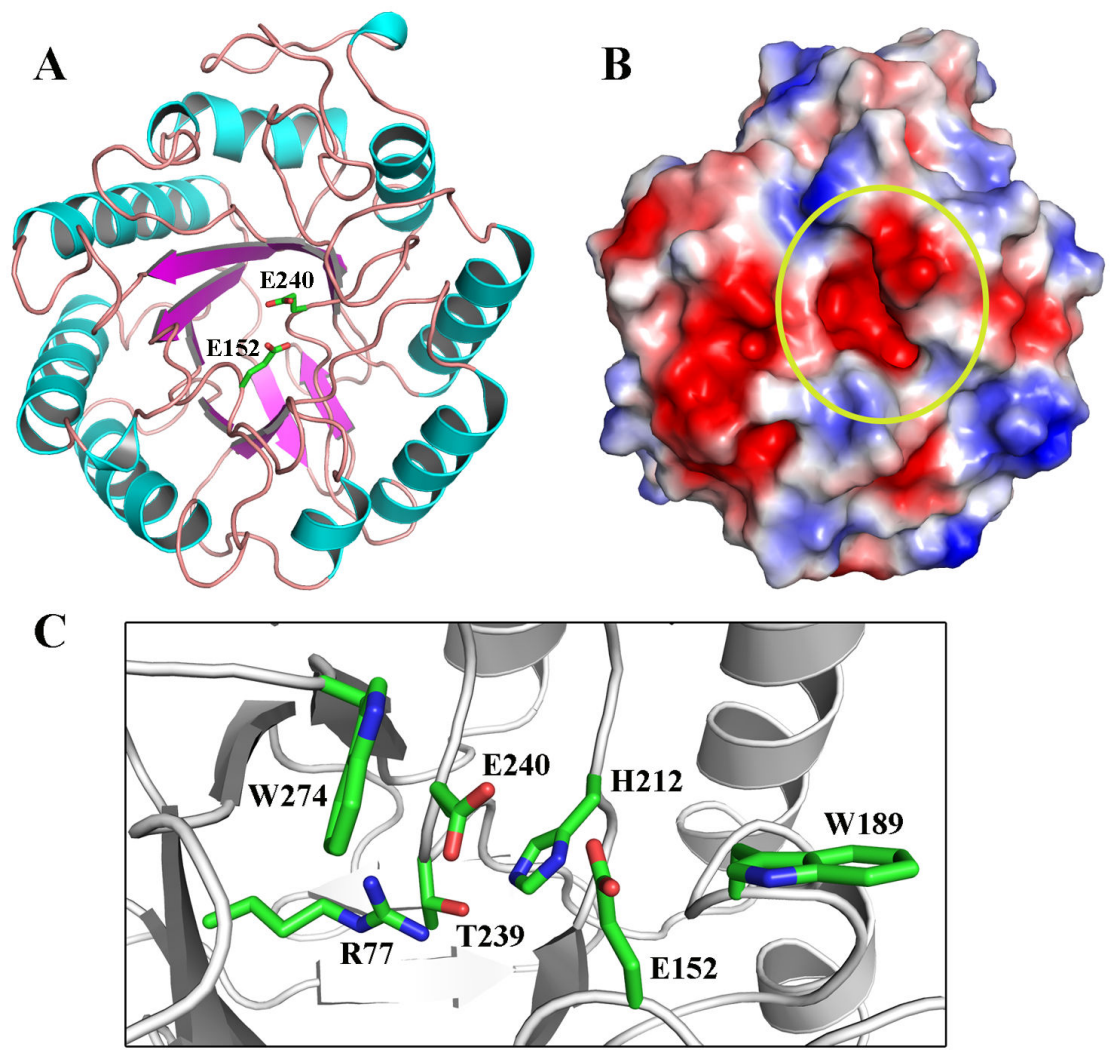
Structural studies of CelE1. (A) Overall structure of the CelE1 showing a classical (β/α)_8_-barrel fold with the two catalytic acidic residues depicted. (B) Surface charge distribution with highlight to highly negatively charged active-site pocket that is essential for substrate binding and cleavage. (C) Details of the active site in which catalytically-relevant residues are indicated.

 The final model converged to a R_work_/R_free_ of 13.33/17.34% with excellent global and local stereochemical properties (Table 1 and Table S1 in File S1). The enzyme displays a canonical (β/α)_8_-barrel fold (also known as TIM-barrel) characteristic for members of GH5 family, which is composed by eight β-sheets surrounded by eight α-helices (Figure 4A). This structural scaffold is extremely versatile, being able to harbor a number of activities related or not to glycoside hydrolases [34]. The long loops connecting the C-terminal end of β-strands with the N-termini of the external layer of helices delineate the negatively charged catalytic cleft (Figure 4B). The opposite side of the active site contains an extra anti-parallel β-sheet, commonly present in cellulases 5 that contributes to conformational stability [23,35-38].

By comparison with other cellulases the residues Glu^152^ and Glu^240^ were identified as the catalytic acidic residues, where Glu^152^ is the proton donor and Glu^240^ the nucleophile. These residues are separated by a distance of 6.1 Å, compatible for a retaining catalytic mechanism (Figure 4C). Other important residues for catalysis are conserved including His^117^ and His^212^ [25,35,39,40] and the aromatic gatekeepers Trp^189^ and Trp^241^ (Figure 4C). These tryptophan residues play a key role in sugar binding through aromatic stacking interactions with the glucopyranosyl rings [41].

### The extended β8-α8 loop generates additional intramolecular contacts that might contribute to CelE1 thermal stability

**Figure 5:**
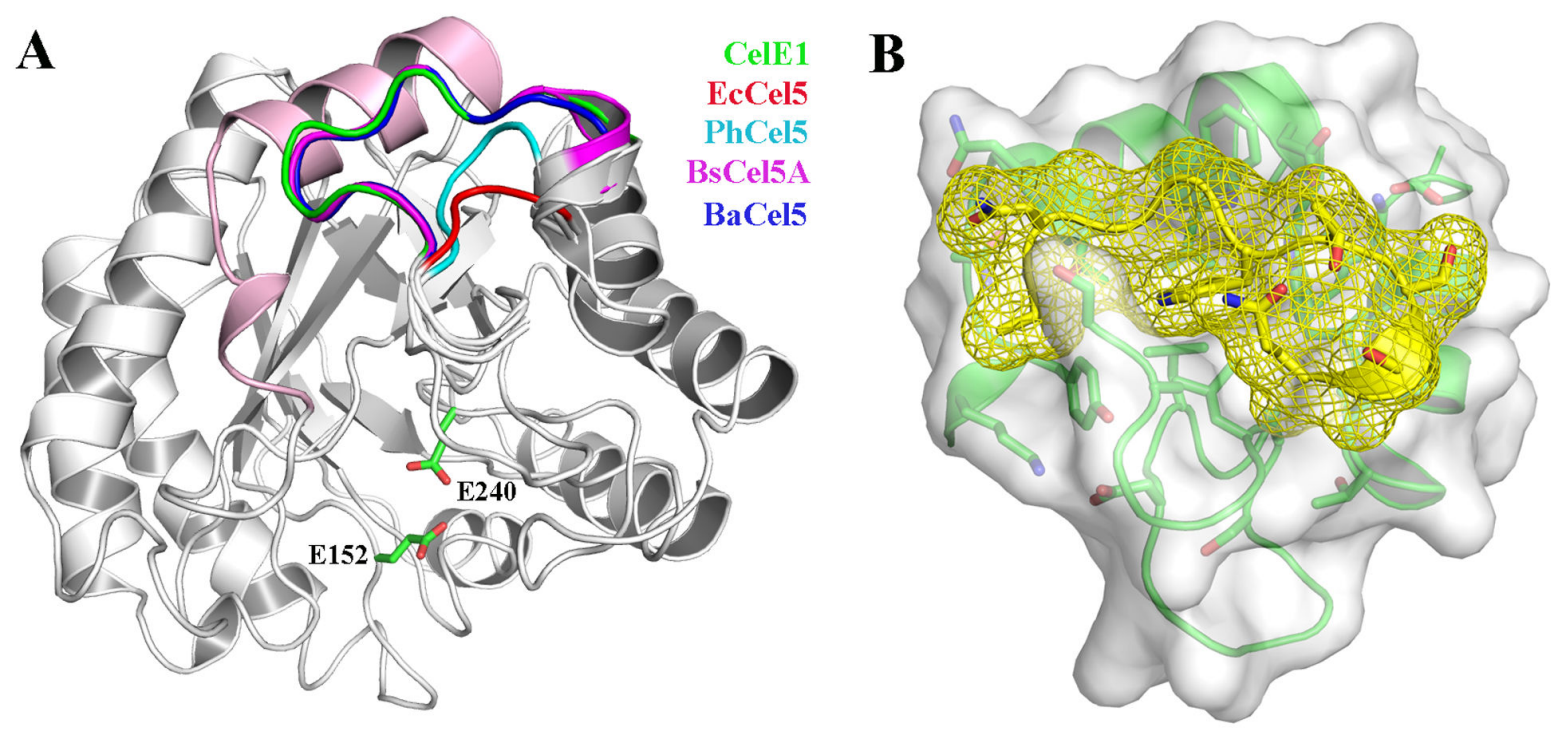
Comparative structural analysis of CelE1 (4M1R) with other structurally similar cellulases 5. (A) Representation of the extended α_8_/β_8_ loop conserved in thermostable enzymes (BsCel5A, *Bacillus subtilis*, 3PZU; BaCel5A, *Bacillus agaradhaerens*, 1QHZ) in comparison to meso- and psychrophilic cellulases (EcCel5, *Erwinia chrysanthemi*, 1EGZ; Cel5G, *Pseudoalteromonas haloplanktis*, 1TVN). The helix α1 that makes new interactions with the extended α_8_/β_8_ loop is colored in light pink. (B) Surface complementarity between the extended α_8_/β_8_ loop (yellow mesh) and the neighboring structural elements (green) indicating the additional intramolecular contacts favored by this motif.

CelE1 is structurally similar to other characterized GH5 cellulases with RMSD values for Cα atoms of 0.39, 0.43, 0.56 and 0.64 Å in relation to cellulases from *Erwinia chrysanthemi* [35], *Pseudoalteromonas haloplanktis* [18], *Bacillus agaradhaerens* [42] and *Bacillus subtillis* [23], respectively. Despite the fully conserved catalytic cleft, some significant structural differences were found in the interfacial loops that vary in length and composition (Figure 5A and Figure S3 in File S1). In contrast to the cellulase from *Erwinia chrysanthemi* (EcCel5, PDB code 1EGZ) and its psychrophilic ortholog Cel5G from *Pseudoalteromonas haloplanktis* (PDB code 1TVN), CelE1 contains an insertion of eight residues in the β8-α8 loop (^292^ANGGWTSS^299^). This extended loop promotes additional intramolecular contacts that might be related to the significant higher structural stability of CelE1 in comparison to the Cel5G from *Pseudoalteromonas haloplanktis* and other psychrophilic orthologs (Figure 5B). Moreover, other cellulases 5 with similar thermal stability as those isolated from *B. subtilis* (BsCel5A, Tm ~60°C) [23] and *B. agaradhaerens* (BaCel5, PDB code 1QHZ) [42] also have an equivalent insertion in the β8-α8 loop, supporting our finding (Figure 5B). The comparison with both BsCel5A and BaCel5 reveals other small insertions or deletions in interfacial loops (Figure S3 in File S1), but these regions are not implicated in generating new intramolecular contacts as those observed by the 8-residue-long insertion in the β8-α8 loop for CelE1. These analyses suggest that additional residues in the β8-α8 loop might contribute to stabilize the structure improving the thermal tolerance of cellulases.

## Conclusion

A new plant cell wall-degrading enzyme with ability to breakdown complex cellulose-based substrates was isolated and characterized from sugarcane soil metagenome. This enzyme was shown to be an endo-acting glucanase with high catalytic activity at a broad temperature range and under alkaline conditions that usually cause enzyme inactivation of classical acidic cellulases. Moreover, its crystal structure was determined at 1.8 Å resolution indicating that the 8-residue-long insertion in the β8-α8 loop might confer higher conformational stability in comparison to its psychrophilic orthologs. This work contributes to both basic and applied knowledge of cellulases and provides a promising biocatalyst with unusual biochemical properties for industrial processes involving degradation of lignocellulosic materials.

## Supporting Information

File S1 **Biophysical and structural analyses of CelE1.**
(PDF)

## References

[1] Lorenz P, Eck J (2005) Metagenomics and industrial applications. Nat Rev Microbiol 3: 510-516. doi:10.1038/nrmicro1161. PubMed: 15931168. 15931168

[2] Farrell AE, Plevin RJ, Turner BT, Jones AD, O'Hare M et al. (2006) Ethanol can contribute to energy and environmental goals. Science 311: 506-508. doi:10.1126/science.1121416. PubMed: 16439656. 16439656

[3] Margeot A, Hahn-Hagerdal B, Edlund M, Slade R, Monot F (2009) New improvements for lignocellulosic ethanol. Curr Opin Biotechnol 20: 372-380. doi:10.1016/j.copbio.2009.05.009. PubMed: 19502048. 19502048

[4] Wen F, Nair NU, Zhao H (2009) Protein engineering in designing tailored enzymes and microorganisms for biofuels production. Curr Opin Biotechnol 20: 412-419. doi:10.1016/j.copbio.2009.07.001. PubMed: 19660930. 19660930 PMC2763986

[5] Festucci-Buselli RA, Otoni WC, Joshi CP (2007) Structure, organization, and functions of cellulose synthase complexes in higher plants. Braz J Plant Physiol 19: 1-13.

[6] Pereira JH, Sapra R, Volponi JV, Kozina CL, Simmons B et al. (2009) Structure of endoglucanase Cel9A from the thermoacidophilic Alicyclobacillus acidocaldarius. Acta Crystallogr D Biol Crystallogr 65: 744-750. doi:10.1107/S0907444909012773. PubMed: 19622857. 19622857 PMC2714717

[7] Henrissat B, Bairoch A (1993) New families in the classification of glycosyl hydrolases based on amino acid sequence similarities. Biochem J 293 ( 3): 781-788. PubMed: 8352747. 8352747 10.1042/bj2930781 PMC1134435

[8] Alvarez TM, Goldbeck R, Dos Santos CR, Paixão DA, Gonçalves TA et al. (2013) Development and Biotechnological Application of a Novel Endoxylanase Family GH10 Identified from Sugarcane Soil Metagenome. PLOS ONE, 8: e70014 PubMed: 23922891. 23922891 10.1371/journal.pone.0070014 PMC3726488

[9] Teather RM, Wood PJ (1982) Use of Congo red-polysaccharide interactions in enumeration and characterization of cellulolytic bacteria from the bovine rumen. Appl Environ Microbiol 43: 777-780. PubMed: 7081984. 7081984 10.1128/aem.43.4.777-780.1982 PMC241917

[10] Laemmli UK (1970) Cleavage of structural proteins during the assembly of the head of bacteriophage T4. Nature 227: 680-685. doi:10.1038/227680a0. PubMed: 5432063. 5432063

[11] Miller GL (1959) Use of dinitrosalicylic acid reagent for determination of reducing sugar. Anal Chem 31: 426-428. doi:10.1021/ac60147a030.

[12] McIlvaine TC (1921) A buffer solution for colorimetric comparison. J Biol Chem 49: 183 - 186.

[13] Cota J, Alvarez TM, Citadini AP, Santos CR, de Oliveira Neto M et al. (2011) Mode of operation and low-resolution structure of a multi-domain and hyperthermophilic endo-beta-1,3-glucanase from Thermotoga petrophila. Biochem Biophys Res Commun 406: 590-594. doi:10.1016/j.bbrc.2011.02.098. PubMed: 21352806. 21352806

[14] Whitmore L, Wallace BA (2004) DICHROWEB, an online server for protein secondary structure analyses from circular dichroism spectroscopic data. Nucleic Acids Res 32: W668-W673. doi:10.1093/nar/gkh371. PubMed: 15215473. 15215473 PMC441509

[15] Whitmore L, Wallace BA (2008) Protein secondary structure analyses from circular dichroism spectroscopy: methods and reference databases. Biopolymers 89: 392-400. doi:10.1002/bip.20853. PubMed: 17896349. 17896349

[16] Matthews BW (1968) Solvent content of protein crystals. J Mol Biol 33: 491-497. doi:10.1016/0022-2836(68)90205-2. PubMed: 5700707. 5700707

[17] Vagin A, Teplyakov A (2010) Molecular replacement with MOLREP. Acta Crystallogr D Biol Crystallogr 66: 22-25. doi:10.1107/S0907444909042589. PubMed: 20057045. 20057045

[18] Violot S, Aghajari N, Czjzek M, Feller G, Sonan GK et al. (2005) Structure of a full length psychrophilic cellulase from *Pseudoalteromonas* *haloplanktis* revealed by X-ray diffraction and small angle X-ray scattering. J Mol Biol 348: 1211-1224. doi:10.1016/j.jmb.2005.03.026. PubMed: 15854656. 15854656

[19] Murshudov GN, Skubák P, Lebedev AA, Pannu NS, Steiner RA et al. (2011) REFMAC5 for the refinement of macromolecular crystal structures. Acta Crystallogr D Biol Crystallogr 67: 355-367. doi:10.1107/S0907444911001314. PubMed: 21460454. 21460454 PMC3069751

[20] Emsley P, Cowtan K (2004) Coot: model-building tools for molecular graphics. Acta Crystallogr D Biol Crystallogr 60: 2126-2132. doi:10.1107/S0907444904019158. PubMed: 15572765. 15572765

[21] Park JI, Kent MS, Datta S, Holmes BM, Huang Z et al. (2011) Enzymatic hydrolysis of cellulose by the cellobiohydrolase domain of CelB from the hyperthermophilic bacterium Caldicellulosiruptor saccharolyticus. Bioresour Technol 102: 5988-5994. doi:10.1016/j.biortech.2011.02.036. PubMed: 21421309. 21421309

[22] Liu J, Liu WD, Zhao XL, Shen WJ, Cao H et al. (2011) Cloning and functional characterization of a novel endo-beta-1,4-glucanase gene from a soil-derived metagenomic library. Appl Microbiol Biotechnol 89: 1083-1092. doi:10.1007/s00253-010-2828-4. PubMed: 20938774. 20938774

[23] Santos CR, Paiva JH, Sforça ML, Neves JL, Navarro RZ et al. (2012) Dissecting structure-function-stability relationships of a thermostable GH5-CBM3 cellulase from *Bacillus* *subtilis* 168. Biochem J 441: 95-104. doi:10.1042/BJ20110869. PubMed: 21880019. 21880019

[24] Nguyen NH, Maruset L, Uengwetwanit T, Mhuantong W, Harnpicharnchai P et al. (2012) Identification and characterization of a cellulase-encoding gene from the buffalo rumen metagenomic library. Biosci Biotechnol Biochem 76: 1075-1084. PubMed: 22790926. 22790926 10.1271/bbb.110786

[25] Ducros V, Czjzek M, Belaich A, Gaudin C, Fierobe HP et al. (1995) Crystal structure of the catalytic domain of a bacterial cellulase belonging to family 5. Structure 3: 939-949. doi:10.1016/S0969-2126(01)00228-3. PubMed: 8535787. 8535787

[26] Bischoff KM, Rooney AP, Li XL, Liu S, Hughes SR (2006) Purification and characterization of a family 5 endoglucanase from a moderately thermophilic strain of Bacillus licheniformis. Biotechnol Lett 28: 1761-1765. doi:10.1007/s10529-006-9153-0. PubMed: 16900329. 16900329

[27] Liang C, Xue Y, Fioroni M, Rodríguez-Ropero F, Zhou C et al. (2011) Cloning and characterization of a thermostable and halo-tolerant endoglucanase from *Thermoanaerobacter* *tengcongensis* MB4. Appl Microbiol Biotechnol 89: 315-326. doi:10.1007/s00253-010-2842-6. PubMed: 20803139. 20803139

[28] Badieyan S, Bevan DR, Zhang C (2012) A salt-bridge controlled by ligand binding modulates the hydrolysis reaction in a GH5 endoglucanase. Protein Eng Des Sel 25: 223-233. doi:10.1093/protein/gzs010. PubMed: 22419828. 22419828

[29] Barras F, Bortoli-German I, Bauzan M, Rouvier J, Gey C et al. (1992) Stereochemistry of the hydrolysis reaction catalyzed by endoglucanase Z from *Erwinia* *chrysanthemi* . FEBS Lett 300: 145-148. doi:10.1016/0014-5793(92)80183-H. PubMed: 1563515. 1563515

[30] Cohen R, Suzuki MR, Hammel KE (2005) Processive endoglucanase active in crystalline cellulose hydrolysis by the brown rot basidiomycete *Gloeophyllum* *trabeum* . Appl Environ Microbiol 71: 2412-2417. doi:10.1128/AEM.71.5.2412-2417.2005. PubMed: 15870328. 15870328 PMC1087581

[31] Franco Cairo JP, Oliveira LC, Uchima CA, Alvarez TM, Citadini AP et al. (2013) Deciphering the synergism of endogenous glycoside hydrolase families 1 and 9 from *Coptotermes* *gestroi* . Insect Biochem Mol Biol 43: 970-981. doi:10.1016/j.ibmb.2013.07.007. PubMed: 23917163. 23917163

[32] Sonan GK, Receveur-Brechot V, Duez C, Aghajari N, Czjzek M, Haser R, Gerday C (2007) The linker region plays a key role in the adaptation to cold of the cellulase from an Antarctic bacterium. Biochem J 407: 293-302. doi:10.1042/BJ20070640. PubMed: 17635108. 17635108 PMC2049020

[33] Krissinel E, Henrick K (2007) Inference of macromolecular assemblies from crystalline state. J Mol Biol 372: 774-797. doi:10.1016/j.jmb.2007.05.022. PubMed: 17681537. 17681537

[34] Brändén C-I (1991) The TIM barrel-the most frequently occurring folding motif in proteins. Curr Opin Struct Biol 1: 978–983. doi:10.1016/0959-440X(91)90094-A.

[35] Chapon V, Czjzek M, El Hassouni M, Py B, Juy M et al. (2001) Type II protein secretion in gram-negative pathogenic bacteria: the study of the structure/secretion relationships of the cellulase Cel5 (formerly EGZ) from *Erwinia* *chrysanthemi* . J Mol Biol 310: 1055-1066. doi:10.1006/jmbi.2001.4787. PubMed: 11501995. 11501995

[36] Shaw A, Bott R, Vonrhein C, Bricogne G, Power S et al. (2002) A novel combination of two classic catalytic schemes. J Mol Biol 320: 303-309. doi:10.1016/S0022-2836(02)00387-X. PubMed: 12079387. 12079387

[37] Davies GJ, Dauter M, Brzozowski AM, Bjørnvad ME, Andersen KV et al. (1998) Structure of the *Bacillus* *agaradherans* family 5 endoglucanase at 1.6 A and its cellobiose complex at 2.0 A resolution. Biochemistry 37: 1926-1932. doi:10.1021/bi972162m. PubMed: 9485319. 9485319

[38] Shirai T, Ishida H, Noda J, Yamane T, Ozaki K et al. (2001) Crystal structure of alkaline cellulase K: insight into the alkaline adaptation of an industrial enzyme. J Mol Biol 310: 1079-1087. doi:10.1006/jmbi.2001.4835. PubMed: 11501997. 11501997

[39] Dominguez R, Souchon H, Spinelli S, Dauter Z, Wilson KS et al. (1995) A common protein fold and similar active site in two distinct families of beta-glycanases. Nat Struct Biol 2: 569-576. doi:10.1038/nsb0795-569. PubMed: 7664125. 7664125

[40] Sakon J, Adney WS, Himmel ME, Thomas SR, Karplus PA (1996) Crystal structure of thermostable family 5 endocellulase E1 from *Acidothermus* *cellulolyticus* in complex with cellotetraose. Biochemistry 35: 10648-10660. doi:10.1021/bi9604439. PubMed: 8718854. 8718854

[41] Vyas NK (1991) Atomic features of protein-carbohydrate interactions. Curr Opin Struct Biol 1: 723-740.

[42] Varrot A, Schülein M, Davies GJ (2000) Insights into ligand-induced conformational change in Cel5A from *Bacillus* *agaradhaerens* revealed by a catalytically active crystal form. J Mol Biol 297: 819-828. doi:10.1006/jmbi.2000.3567. PubMed: 10731432. 10731432

