# Supplementary material for: Structure and Function of a Novel Cellulase 5 from Sugarcane Soil Metagenome
Source: PLoS One. 2013 Dec 17;8(12):e83635. doi: 10.1371/journal.pone.0083635 (PMC3866126; doi:10.1371/journal.pone.0083635)
Supplement: File S1 — Biophysical and structural analyses of CelE1. (PDF) [file pone.0083635.s001.pdf]

Supporting information

Structure and function of a novel cellulase 5 from sugarcane soil metagenome

Thabata M. Alvarez[1,#], Joice H. Paiva[2,#], Diego M. Ruiz[2], João P. L. F. Cairo[1], Isabela O. Pereira[1], Douglas A. A. Paixão[1], Rodrigo F. de Almeida[1], Celisa C. C. Tonoli[2], Roberto Ruller[1], Camila R. Santos[2], Fabio M. Squina[1,*], Mario T. Murakami[2,*]

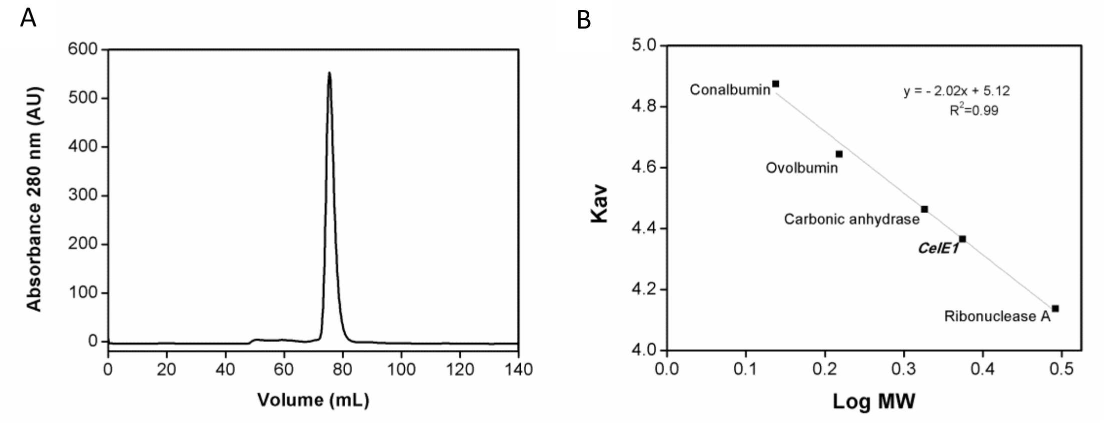

Figure S1: Size-exclusion chromatography of CelE1. (A) Elution profile of CelE1 on a Superdex 75 16/60 column using 20 mM phosphate buffer at pH 7.4 containing 150 mM NaCl at 0.5 mL/min. (B) Calibration curve with known standard proteins indicating a monomeric state of CelE1.

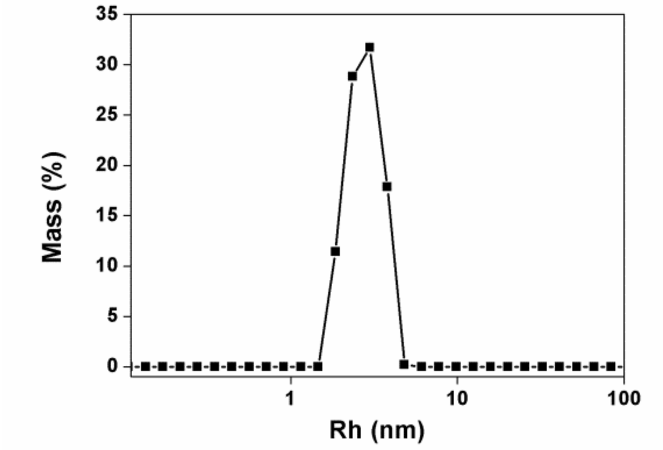

Figure S2: Dynamic light scattering analysis of the purified CelE1 indicating a monomodal particle size distribution with a hydrodynamic radius of 2.8 nm and polydispersity of 20.6%.

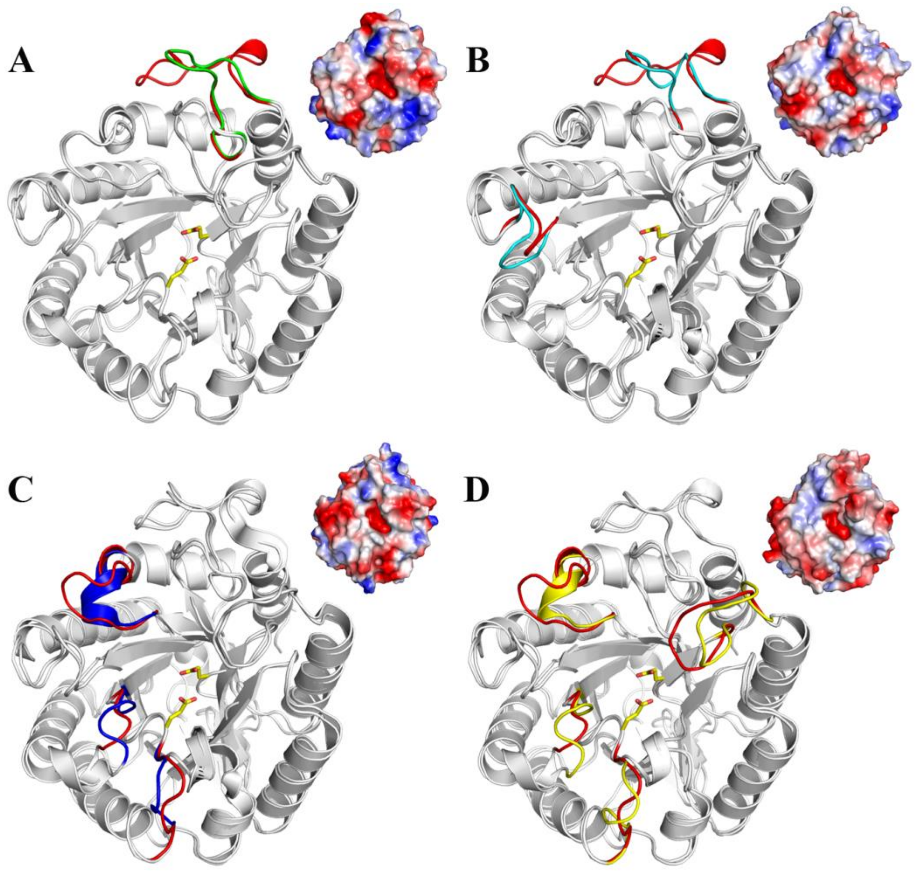

Figure S3: Comparative structural analysis of CelE1 (4M1R) with other structurally characterized cellulases 5. (A). EcCel5, *Erwinia chrysanthemi* (1EGZ). (B) Cel5G, *Pseudoalteromonas haloplanktis* (1TVN). (C) BsCel5A, *Bacillus subtilis* (3PZU). (D) BaCel5A, *Bacillus agaradhaerens* (1QHZ). Superposed structures are shown as gray ribbon diagrams including residues involved in catalysis (yellow carbon atoms). The distinct loops are colored in red (CelE1), green (EcCel5), cyan (Cel5G), blue (BsCel5A) and yellow (BaCel5A).

Table S1: Summary statistics of CelE1 structure according to MolProbity analysis.

Protein Geometry	Poor rotamers	3	0.64%	Goal: <1%
	Ramachandran outliers	0	0.00%	Goal: <0.05%
	Ramachandran favored	566	96.26%	Goal: >98%
	Cβ deviations >0.25Å	0	0.00%	Goal: 0
	Bad backbone bonds:	0 / 2366	0.00%	Goal: 0%
	Bad backbone angles:	0 / 2954	0.00%	Goal: <0.1%
